# Optimal Waist-to-Hip Ratios in Women Activate Neural Reward Centers in Men

**DOI:** 10.1371/journal.pone.0009042

**Published:** 2010-02-05

**Authors:** Steven M. Platek, Devendra Singh

**Affiliations:** 1 Department of Psychology, Georgia Gwinnett College, Lawrenceville, Georgia, United States of America; 2 Department of Psychology, University of Texas at Austin, Austin, Texas, United States of America; University of Utah, United States of America

## Abstract

Secondary sexual characteristics convey information about reproductive potential. In the same way that facial symmetry and masculinity, and shoulder-to-hip ratio convey information about reproductive/genetic quality in males, waist-to-hip-ratio (WHR) is a phenotypic cue to fertility, fecundity, neurodevelopmental resources in offspring, and overall health, and is indicative of “good genes” in women. Here, using fMRI, we found that males show activation in brain reward centers in response to naked female bodies when surgically altered to express an optimal (∼0.7) WHR with redistributed body fat, but relatively unaffected body mass index (BMI). Relative to presurgical bodies, brain activation to postsurgical bodies was observed in bilateral orbital frontal cortex. While changes in BMI only revealed activation in visual brain substrates, changes in WHR revealed activation in the anterior cingulate cortex, an area associated with reward processing and decision-making. When regressing ratings of attractiveness on brain activation, we observed activation in forebrain substrates, notably the nucleus accumbens, a forebrain nucleus highly involved in reward processes. These findings suggest that an hourglass figure (i.e., an optimal WHR) activates brain centers that drive appetitive sociality/attention toward females that represent the highest-quality reproductive partners. This is the first description of a neural correlate implicating WHR as a putative honest biological signal of female reproductive viability and its effects on men's neurological processing.

## Introduction

Variations in men's facial (e.g., symmetry, masculinity) and body (e.g., shoulder-to-hip ratio) morphology are related to women's ratings of attractiveness. Specifically, women tend to rate more symmetrical and masculine faces, and higher SHR[1] as attractive during fertile phases of their menstrual cycle and for short-term mating partners[2], [3], [4], [5], [6], [7], [8]. Several recent findings[9], [10], [11], [12] demonstrate that faces that are rated as attractive activate neural reward substrates in females, and that this neural activation is partly modulated by hormonal status of women[11]. These findings support the hypothesis that men's facial and body morphology serves as an honest biosignal to genetic fitness.

Women have a larger proportion of fat stores in comparison to non-human female primates and men[13]. Prior to puberty, a sexual dimorphism in fat distribution is not apparent, however, in post-menarche women estrogen inhibits storage of fat around abdominal areas and fat is stored in the gluteofemoral (thighs and buttock) region[14]. Women have a lower waist-to-hip ratio (WHR) than men and men's preference for lower WHR, or hour-glass figures, appears to be cross-cultural[15], [16]c.f.[17], [18] and adaptive because WHR is positively correlated with fecundity, levels of the reproductive hormones estradiol and progesterone[19], higher likelihood of conception[20], and availability of neurodevelopmental resources for offspring[21], but negatively correlated with lower likelihood of illnesses such as heart disease and various cancers[15], and lower incidence of depression[22]. Differences in WHR can also be used to index pregnancy and the capacity for unencumbered childbirth[1]. Consequently, it's been hypothesized that distribution of body fat represents a secondary sexual characteristic that, in the same way variation in men's facial and body morphology does, can influence ratings of attraction and fecundity (see also[23] for arguments regarding female BMI).

## Methods

### Participants

Here 14 men (*M_age_ = 25.21, S.D. = 6.30*) were scanned using fMRI while making attractiveness ratings to randomly and individually presented pictures (one from the rear and one from an oblique rear position for each woman) of seven naked female bodies prior to and after recovery from an elective cosmetic surgical procedure to reconfigure and optimize WHR[14]. All participants provided written informed consent and the study was approved by the University of Liverpool School of Biological Sciences committee on research ethics.

### Stimuli

Stimuli were a sample of seven images taken from those used in another study [14]. Images were presented in both the rear and oblique rear position (see [14]). Images were presented 15 times for 1 second with a variable interstimulus (2–15 s) interval to maximize jittering using Neurobehavioral Systems Presentation. Each participant saw each of the 14 different images in random order. Participants were asked to respond to the attractiveness of the images by using a 5 button fMRI-comaptible response pad (MR technologies). Order of button anchor (most attractive/least attractive) was randomized across subjects.

### fMRI Procedures

Data were collected using a Siemens Trio 3T scanner using gradient-echo T2*-weighted echo-planar images (EPI) to measure blood-oxygen-level-dependent (BOLD) contrasts. For each participant approximately 225 volumes were collected with an interleaved acquisition, a slice thickness of 2 mm, at a TR of 2.75 s, TE of 30 ms, and a resolution of 3.5×3.5 mm for each participant. Slices were tilted to 30 degrees from horizontal to maximize image quality in areas near susceptibility field gradients[24]. All participants also underwent high-resolution structural scanning with a standard MP-RAGE sequence (176 1 mm voxel saggital slices, TR = 2040 ms, TE = 5.57 ms, FoV = 256, flip angle = 8°). While being scanned participants were asked to make explicit ratings of attractiveness to naked female body images [14] presented for 1 second (ISI randomization optimized for jitter 2–15 seconds). Ratings were made on a 5-point scale from very unattractive to very attractive (counterbalanced for which finger anchored each end of the attractiveness scale).

fMRI data were analyzed using general linear modeling and mixed effects analysis implemented in FEAT-FSL[25]. We hypothesized that if WHR represents a cue to genetic quality then, similar to symmetry and masculinity in male faces, bodies that represent optimal WHR would activate neural reward substrates.

## Results

**Figure 1:**
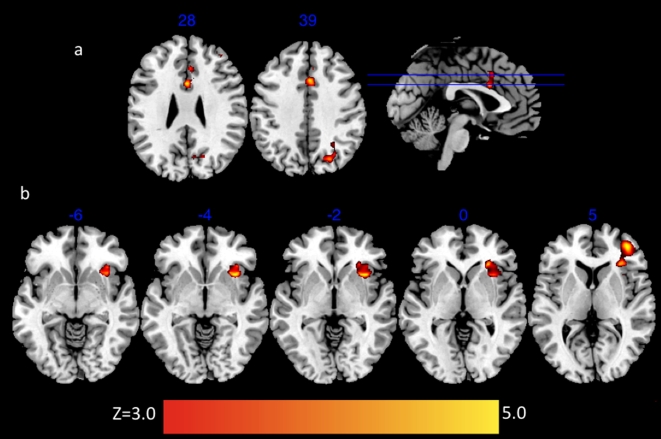
Activation associated with pre-surgical minus post-surgical contrast. (a) Statistical parametric map for contrast post-surgical versus pre-surgical bodies showing activation in anterior cingulate cortex and (c) right orbital frontal cortex at cluster corrected threshold of 3.0, p<.01.

**Figure 2:**
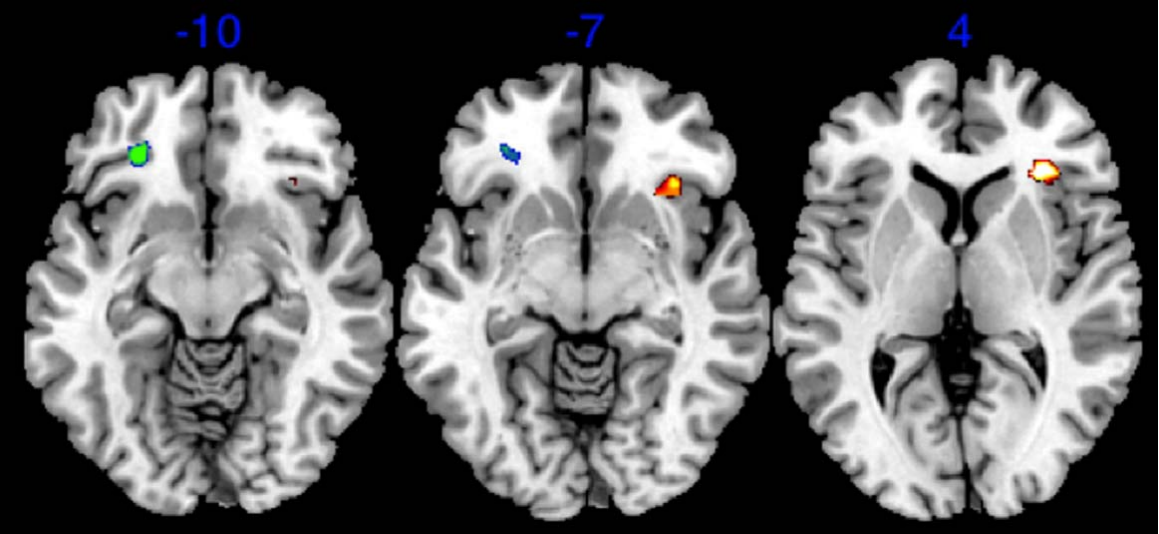
Statistical parametric map showing activation in bilateral OFC region of interest to post-surgical bodies.

In order to determine whether the WHR surgical procedure had an effect on brain activation we first computed a contrast between pre-surgical and post-surgical bodies. Relative to viewing pre-surgical bodies, viewing post-surgical bodies revealed activation in right orbital frontal cortex (OFC) (*Z = 4.11, p<.01 cluster corrected*), lateral occipital cortex (*Z = 3.82, p<.01 cluster corrected*), and the anterior cingulate gyrus (*Z = 3.71, p<.01*) (Fig. 1a–b). Because the OFC has been associated with reward evaluation we created a bilateral OFC region of interest (ROI) mask to investigate the extent of activation in this area specifically. This analysis revealed that post-surgical images activated the left (*Z = 3.59, p<.01*) and the right (*Z = 3.77, p<.01*) OFC, but pre-surgical images did not (Fig. 2). An ROI of activation in bilateral amygdala, another area often associated with reward processing[26], [27], [28], [29] and recently associated with implicit social judgment making[30], [31] revealed no voxels survived statistical threshold for pre- or post-surgical bodies. This finding demonstrates that changes associated with cosmetic surgery (aimed at optimizing female WHR) have specific effects on men's OFC, an area of the brain that is associated with evaluations of rewards.

**Figure 3:**
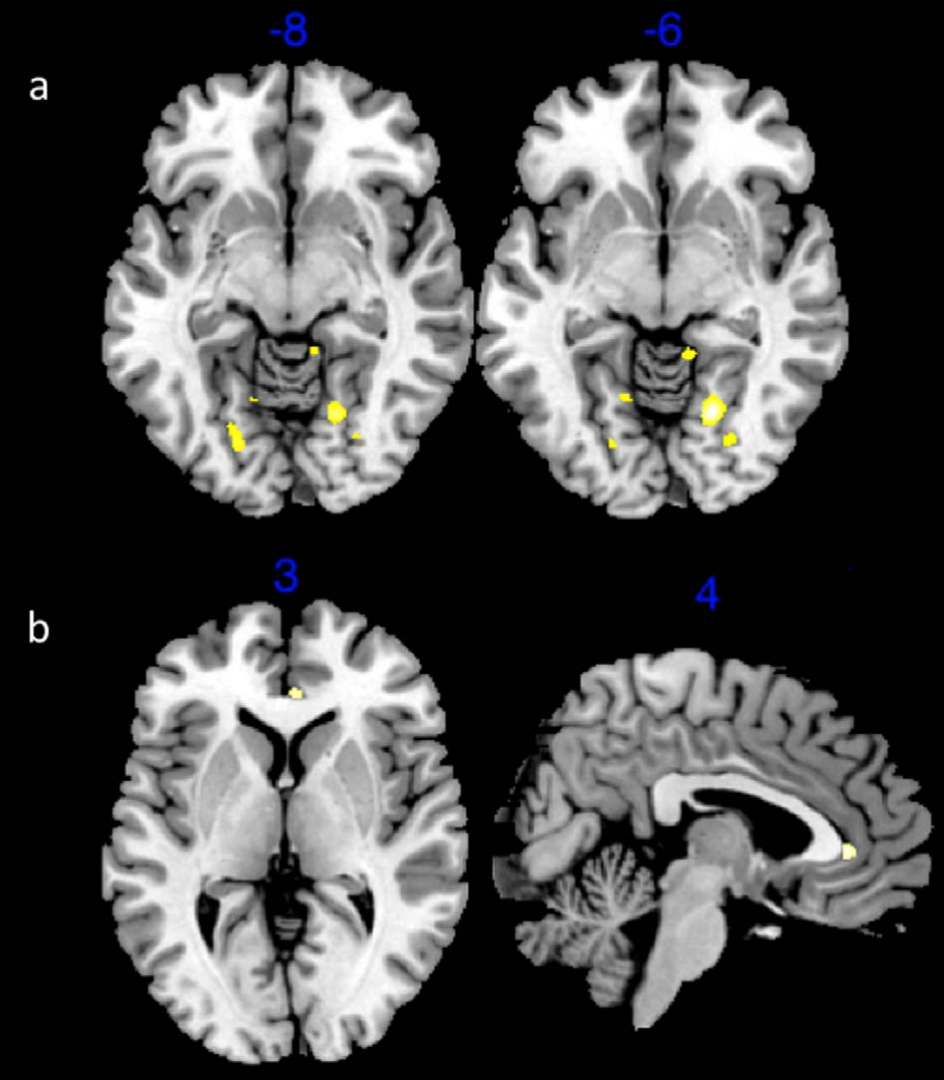
Activation associated with change in BMI and WHR, respectively. (a) Statistical parametric map showing activation in visual areas to changes in BMI. (b) Activation to changes in WHR in the anterior paracingulate gyrus. (parametric analysis, p<.001 uncorrected).

We then regressed each woman's change in BMI and WHR between pre-surgery and post-surgery onto the participants' brain activation to determine how changes in BMI and WHR differentially affected the brains of men. While changes in BMI only revealed activation in visual brain substrates (right lingual gyrus *Z = 2.72, p<.001 uncorrected*; left fusiform gyrus *Z = 2.25, p<.001 uncorrected*; and right lateral occipital cortex *Z = 2.21, p<.001 uncorrected*) (Fig. 3a), changes in WHR revealed activation in the right para-anterior cingulate gyrus (*Z = 2.58, p<.001 uncorrected*) (Fig. 3b). This finding suggests that changes in BMI activate low-level visual areas that are tuned to noticing variations in body configuration, but not involved in the aesthetic evaluation of the body. On the other hand, changes in WHR activated the anterior paracingulate gyrus, which has been associated with reward processing[26], [29], social evaluation and decision-making[32], [33], and responses under uncertainty and adaptive personal significance[34].

**Figure 4:**
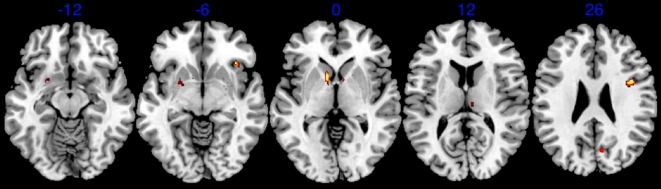
Activation as a function of attractiveness ratings. Statistical parametric map showing activation when attractiveness ratings were regressed on post-surgical images of bodies. (parametric analysis, p<.001 uncorrected).

Lastly, we regressed each participant's idiosyncratic ratings of attractiveness on their brain activation to post- minus pre-surgical bodies to investigate how explicit ratings of body attractiveness predict brain activation. This revealed activation in various forebrain structures including the left (*Z = 3.40, p<.001 uncorrected*) and right (*Z = 4.30, p<.001 uncorrected*) OFC, left putamen (*Z = 3.70, p<.001 uncorrected*), left nucleus accumbens (*Z = 4.22, p<.001 uncorrected*), left (*Z = 4.93, p<.001 uncorrected*), right (*Z = 3.90, p<.001 uncorrected*) caudate, and right thalamus (*Z = 3.47, p<.001 uncorrected*) (Fig. 4). This activation pattern, particularly activation of the nucleus accumbens, suggests that attractiveness ratings were associated with activation in neural reward centers[9], [10], [26], [27], [28], [35], [36], [37], [38], [39], [40] that have also been associated with drug/alcohol-induced reward^c.f.^ [41], [42], [43].

## Discussion

Our observed activation patterns in the brain suggest that female body configuration represents a salient stimulus to men and that optimal female body configurations activate areas of men's brains that are associated with reward processing and appetitive behaviors. This activation may represent the proximate neural mechanism of attraction to females that express curvaceous body types and also further account for cross-cultural findings showing that optimal WHR (∼.7) as being consistently rated as attractive. Lastly, this finding may extend our understanding of some men's proclivity to develop a preoccupation with stimuli depicting optimally designed women (e.g., pornography)[44], [45]. Interestingly, our findings did not demonstrate that BMI had a large effect on brain activation except in areas associated with simple visual evaluations of shape and size. This does not downplay the importance of BMI in evaluations of female attractiveness, but may suggest that BMI's role in these evaluations is less the product of evolved psychological mechanisms and more the part of culturally driven, or societal based norms and perceptions.

## Acknowledgments

The authors would like to thank Magdalena Babiszewska for her assistance with data collection. The authors would also like to thank Rebecca Burch, J. Mark Davis, Jennifer Davis, Andrew Gallup, Gordon Gallup, Aaron Goetz, Austen Krill, Katherine Reding, Todd Shackelford, Kyrre Wahtne and the staff at MARIARC for comments on an earlier draft and assistance with fMRI data acquisition.
